# Assessment of Mucormycetes in Subgingival Plaque of Periodontitis and Non-periodontitis Patients: An In Vivo Study

**DOI:** 10.7759/cureus.103725

**Published:** 2026-02-16

**Authors:** Shikha Singh, Arun Kurumathur Vasudevan, Shankar S Menon

**Affiliations:** 1 Periodontics, Amrita School of Dentistry, Amrita Vishwa Vidyapeetham, Kochi, IND

**Keywords:** dental plaque, mucorals, mucormycosis, opportunistic fungal infection, stage iii periodontitis

## Abstract

Background

Periodontitis is a chronic inflammatory disease associated with dysbiotic microbial biofilms. Fungi may also play a role in exacerbating periodontal disease, especially in immunocompromised individuals, where opportunistic infections such as mucormycosis are associated with high morbidity and mortality rates. While mucormycosis occasionally manifests orally, its presence in subgingival plaque, a potential microbial reservoir, remains unexplored. This study investigates whether immunocompetent individuals with periodontitis are at risk for mucormycosis.

Methods

Subgingival plaque was collected from 10 patients with Stage II or III periodontitis and 10 age-matched non-periodontitis controls (aged 18-60 years) at Amrita School of Dentistry’s Department of Periodontics. Exclusion criteria encompassed systemic diseases, recent antibiotic or antifungal use, smoking, pregnancy, and antiseptic mouthwash use within three months. Samples were obtained using sterile Gracey curettes, stored in saline-filled cryovials, and cultured on Sabouraud Dextrose Agar (SDA) at 30°C for 48 hours. Fungal growth was microscopically evaluated for mucormycetes using lactophenol cotton blue staining. The proportion of positive cultures was compared between groups via Pearson’s chi-square test.

Results

None of the samples (n = 20) were identified as containing mucormycetes in either group. No statistics were computed because mucormycosis was constant.

Conclusion

This study found no evidence of mucormycetes in subgingival plaque, indicating that it may not be a common niche in immunocompetent individuals. Larger studies in immunocompromised individuals using molecular approaches are recommended to elucidate fungal roles in periodontal contexts.

## Introduction

Periodontitis ranks among the most prevalent chronic inflammatory diseases globally, affecting nearly 50% of adults and leading to significant tooth loss if untreated. Subgingival plaque, a complex polymicrobial community thriving in an anaerobic niche below the gingival margin, fosters a diverse microbial ecosystem [1]. Etiology centers on a dysbiotic microbial biofilm that disrupts periodontal tissue homeostasis, with bacterial pathogens such as *Porphyromonas gingivalis* (keystone effect), Tannerella forsythia, and Treponema denticola driving inflammation and destruction [1-3]. These organisms appear to be opportunistic pathogens in youths with or without periodontal disease [4]. While bacteria dominate this biofilm, emerging evidence suggests that fungi may also contribute, particularly in states of immune compromise or ecological disruption, such as prolonged antibiotic use or systemic disease [5].

Mucormycosis, a rare yet severe opportunistic fungal infection caused by filamentous fungi of the order Mucorales, class Zygomycetes, poses a significant health threat, with mortality rates often exceeding 50% in disseminated cases [6]. These fungi, including genera such as *Rhizopus*, *Mucor*, and *Lichtheimia*, are ubiquitous saprophytes found in soil, decaying vegetation, and air [7]. The most common cause of mucormycosis in the oral cavity is the transpalatal extension of an existing rhinocerebral infection. Isolated cases of mucormycosis affecting only the gingiva and alveolar bone are extremely uncommon [8]. Infection typically occurs in immunocompromised individuals, those with uncontrolled diabetes, hematologic malignancies, or post-transplant immunosuppression, via inhalation, ingestion, or cutaneous inoculation [9]. Since neutrophils are essential for the host’s defense response, immunological deficiencies linked to hematologic cancer may increase the likelihood of opportunistic fungal infection [10]. Such cases raise the possibility that mucormycetes could colonize oral biofilms such as subgingival plaque, either as transient opportunists or latent reservoirs capable of systemic spread in susceptible hosts [11].

Globally, mucormycosis incidence is rising, partly due to increasing diabetes prevalence and immunosuppressive therapies, with notable surges during the COVID-19 pandemic linked to corticosteroid use [7]. In India, where this study was conducted, mucormycosis is a growing concern, with an estimated prevalence of 140 cases per million, far exceeding rates in developed nations [12]. Oral manifestations, though less common than rhinocerebral forms, underscore the need to investigate mucormycetes’ ecological niches within the mouth [13]. The subgingival plaque’s anaerobic, nutrient-rich environment could theoretically support fungal growth, yet no studies have specifically assessed its role as a mucormycetes reservoir in patients with periodontitis [14].

This study addresses this gap by examining subgingival plaque from patients with Stage II or III periodontitis and non-periodontitis controls. The study explores mucormycetes’ taxonomy, morphology, and environmental prevalence, alongside a literature review of their oral health implications. Mucormycetes are Zygomycetes characterized by broad, nonseptate hyphae and sporangia, distinguishing them from other oral fungi such as Candida [7]. Their environmental ubiquity contrasts with their rarity in healthy hosts, suggesting that host-specific barriers or microbial competition may limit colonization [11]. Documented oral mucormycosis cases, often in immunocompromised patients post-extraction, prompt hypotheses that the subgingival plaque’s polymicrobial nature might harbor these fungi, especially in inflammatory conditions such as periodontitis [9]. This study aims to provide foundational data on the presence of mucormycetes, setting the stage for broader investigations into fungal contributions to periodontal disease.

## Materials and methods

Study design

This prospective cross-sectional in vivo study was conducted in the Department of Periodontics at Amrita School of Dentistry, Kochi, India, following approval from the Institutional Ethics Committee. The study was designed to evaluate the presence of cultivable mucormycetes in subgingival plaque samples collected from patients with periodontitis and periodontally healthy controls under standardized clinical and laboratory conditions.

Ethical considerations

The study was approved by the Institutional Review Board of Amrita School of Medicine, Amrita Institute of Medical Sciences, Kochi, Kerala, India, vide no. ECASM-AIMS-2024-090 dated February 20, 2024. Participants provided written informed consent after receiving detailed explanations of procedures, risks, and benefits.

Participant selection

Twenty participants aged 18-60 years were enrolled: 10 with Stage II or III periodontitis and 10 age-matched non-periodontitis controls. Periodontitis was diagnosed per the 2017 World Workshop classification [15], requiring probing depth (PD) ≥4 mm, interdental clinical attachment loss (CAL) ≥5 mm, and radiographic evidence of alveolar bone loss in at least two nonadjacent teeth [16]. Stage II included CAL of 3-4 mm with PD ≤5 mm, while Stage III involved CAL ≥5 mm with PD ≥6 mm and potential tooth loss due to periodontitis [16]. Controls exhibited PD ≤3 mm, no CAL, and no radiographic bone loss. Exclusion criteria were stringent: systemic diseases (e.g., diabetes, HIV, malignancy), smoking or tobacco use, pregnancy, oral contraceptive use, and antibiotic, antifungal, or antiseptic mouthwash use within three months prior to sampling. These criteria minimized confounders affecting microbial composition or immune status.

Sample collection

Subgingival plaque was collected using sterile Gracey curettes (Hu-Friedy, Chicago, Illinois), designed for precise subgingival access. In periodontitis patients, samples were taken from the deepest pocket per quadrant, targeting sites with PD ≥4 mm. For controls, samples were collected from the mesiobuccal sulcus of first molars, representing healthy sites. Curettes were sterilized via autoclaving (121°C, 15 psi, 15 minutes) and handled with sterile gloves to prevent contamination. Following isolation with cotton rolls and saliva ejectors, the curette was inserted into the pocket or sulcus base, gently scraped upward, and the plaque transferred to 1.5 mL cryovials containing 0.9% sterile saline. Samples were maintained at 4°C and processed within two hours to preserve fungal viability.

Culture and identification

**Figure 1:**
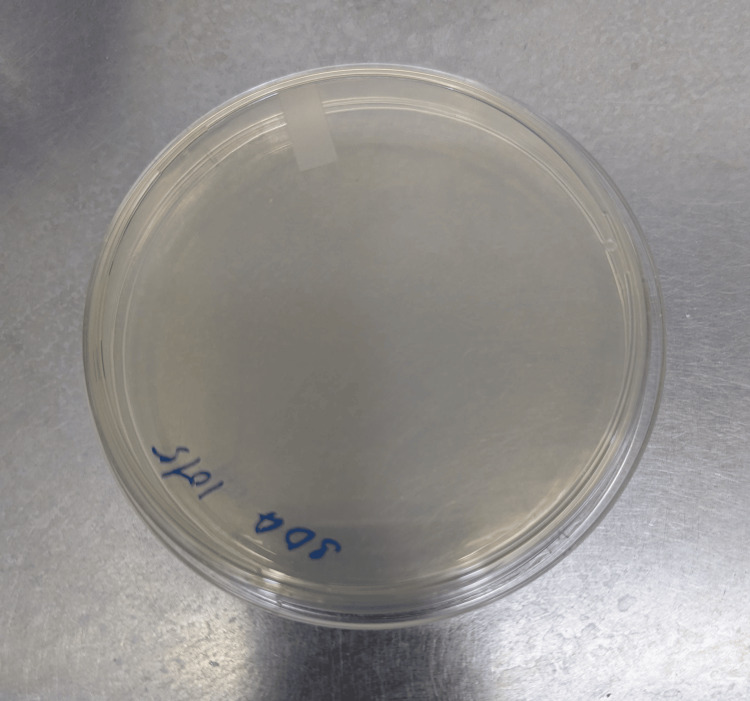
Sabouraud Dextrose Agar medium Figure credits: Dr. Shikha Singh

Samples were irrigated and inoculated onto Sabouraud Dextrose Agar (SDA) plates (HiMedia, Mumbai, India) at 30°C and 35°C. Once good growth was observed, they were subcultured for 48 hours on Sabouraud Dextrose Agar plates, then placed on a VITEK machine (bioMérieux SA, Marcy-l’Étoile, France) for automated identification and on Chrome Agar at 42°C for color identification. Daily visual inspections were performed for identification of fungal colonies, characterized by rapid, cottony growth typical of Mucorales [7]. Suspected colonies underwent wet-mount preparation with lactophenol cotton blue staining and were examined under a light microscope at 40x magnification. Mucormycetes were identified by broad (6-25 µm), nonseptate hyphae with right-angle branching and sporangia, distinguishing them from Candida or Aspergillus [14]. The sample was considered negative if no growth was observed on the SDA slant after four weeks. Figure 1 shows the SDA medium.

Quality control and blinding

Strict aseptic techniques were maintained throughout sample collection and laboratory processing. Culture media were prepared and quality checked according to standard laboratory protocols. Laboratory personnel responsible for microscopic evaluation were blinded to the clinical group allocation of participants to minimize observer bias.

Outcome measures and data handling

The primary outcome measure was the presence or absence of cultivable mucormycetes in subgingival plaque samples. Findings were recorded descriptively and used for comparative analysis between the periodontitis and non-periodontitis groups.

## Results

Subgingival plaque samples from 20 participants (10 with Stage II or III periodontitis, mean age 47.6 ± 11.2 years, 8F/2M; 10 non-periodontitis controls, mean age 36.2 ± 12.8 years, 3F/7M) were analyzed. After inoculation on SDA at 30°C, no mucormycetes were detected in any sample (n = 20). Two periodontitis samples showed non-mucormycetes fungal growth (e.g., Candida-like colonies), which were not further characterized, as the study focused on mucormycetes. Table 1 summarizes case processing, confirming that all 20 samples were valid (100%) with no missing data. The cross-tabulation (Table 2) shows no presence of mucormycetes in either group (10 periodontitis, 10 controls; 50% each in the absent category). Methodological challenges included minor bacterial overgrowth on SDA, mitigated by chloramphenicol, and occasional sample desiccation, which was addressed by saline storage. 

**Table 1:** Case processing summary Table credits: Dr. Shikha Singh

Variable	Cases					
	Valid		Missing		Total	
	N	Percent	N	Percent	N	Percent
Group mucormycosis	20	100.0%	0	0.0%	20	100.0%

**Table 2:** Mucormycosis cross-tabulation Table credits: Dr. Shikha Singh

Group	Variables	Mucormycosis absent	Total
				Non-periodontitis patients	Count	10	10
% within mucormycosis	50.00%	50.00%		
Periodontitis patients	Count	10	10
	% within mucormycosis	50.00%	50.00%
Total	Count	20	20
	% within mucormycosis	100.00%	100.00%

Statistical analysis

Data were recorded as binary (mucormycetes presence or absence). Proportions of positive cultures were compared using Pearson’s chi-square test, with Fisher’s exact test planned for expected frequencies <5 (p < 0.05 threshold). Analysis was performed using IBM SPSS Statistics for Windows, Version 26 (Released 2019; IBM Corp., Armonk, New York). As no mucormycetes were detected (Table 3), mucormycosis was constant (absent), precluding chi-square computation (χ² = 0, p = 1.000). No significant differences existed between groups due to uniformly negative results.

**Table 3:** Chi-square tests ^a^No statistics are computed because mucormycosis is a constant. Table credits: Dr. Shikha Singh

Variables	Value
Pearson chi-square	-^a^
Number of valid cases	20

## Discussion

This study’s failure to detect mucormycetes in subgingival plaque from either periodontitis or non-periodontitis patients suggests that this niche may not commonly harbor these fungi in immunocompetent individuals. This finding aligns with the rarity of mucormycosis in healthy populations, where systemic or local predisposing factors typically facilitate infection [14].

The oral cavity’s innate defenses, including salivary flow, antimicrobial peptides (e.g., histatins), and a balanced microbiome, likely deter mucormycetes colonization. Subgingival plaque, though anaerobic and nutrient-rich, is dominated by bacteria that may competitively exclude fungi [2]. For instance, Porphyromonas gingivalis produces proteases and volatile sulfur compounds that could inhibit fungal growth, a dynamic observed with *Candida albicans* in oral biofilms [5]. Mucormycetes, as environmental saprophytes, thrive in organic-rich substrates but may struggle to adapt to the oral microbiome’s competitive pressures without external triggers such as trauma or immunosuppression [7]. Their broad, nonseptate hyphae require space and oxygen levels that subgingival pockets, despite being hypoxic, may not fully support compared with soil or mucosal surfaces [11].

Host immunity further complicates colonization. In immunocompetent individuals, neutrophils and macrophages effectively phagocytose mucormycetes spores, preventing biofilm integration [14]. Periodontitis, while inflammatory, does not inherently suppress systemic immunity, unlike diabetes or HIV, where mucormycosis flourishes [9]. The polymicrobial nature of subgingival plaque might also favor bacterial-fungal antagonism over synergy, contrasting with *Candida*, a known oral commensal that coexists with bacteria in plaque [5]. Thus, ecological and immunological barriers likely underpin the absence of mucormycetes in this study.

Previous studies have focused on immunocompromised patients, with cases often linked to maxillary sinus invasion post-extraction or trauma [13]. A 2016 case report described an 18-month-old child with oral mucormycosis following malnutrition, highlighting local predispositions [11]. Conversely, *Candida *species are well documented in periodontal pockets, especially in HIV-positive or diabetic patients, suggesting that fungi can colonize subgingival sites under specific conditions [5]. This study’s negative findings contrast with *Candida *prevalence, possibly due to mucormycetes’ lower oral adaptability. Post-COVID-19 mucormycosis surges, predominantly rhinocerebral, reinforce the role of systemic factors (e.g., steroid-induced hyperglycemia) absent in our study [7]. No prior studies have directly assessed mucormycetes in subgingival plaque, making this a novel baseline for comparison.

Limitations

This study has several limitations that should be considered when interpreting the findings. The most important limitation is the small sample size, which limits statistical power and substantially reduces the likelihood of detecting rare organisms such as mucormycetes, particularly in a low-risk, immunocompetent population. Mucormycosis is an uncommon opportunistic infection, and its low prevalence in healthy individuals has been well documented in the literature [7,12]. Consequently, the absence of mucormycetes in this cohort cannot be interpreted as true absence in the general population. In addition, a formal sample size calculation was not performed, as the study was designed as an exploratory investigation intended to generate preliminary baseline data rather than definitive prevalence estimates.

Another significant limitation is the reliance on culture-based methods for fungal detection. Although Sabouraud Dextrose Agar is routinely used for isolating Mucorales, culture techniques have inherent limitations, including low sensitivity and inability to detect nonviable or slow-growing organisms [7,14]. Mucormycetes may be present in low abundance or may require specific environmental conditions that are not fully replicated under standard laboratory incubation, leading to false-negative results. Molecular techniques such as polymerase chain reaction or ribosomal RNA sequencing have been shown to improve detection sensitivity and would be more suitable for identifying rare or fastidious fungi within complex oral biofilms [7,12].

The study population was restricted to immunocompetent individuals, which further limits the generalizability of the findings. Mucormycosis predominantly affects immunocompromised hosts, including individuals with uncontrolled diabetes mellitus, hematologic malignancies, or those receiving corticosteroids or immunosuppressive therapy [9,10,12]. While the inclusion of only immunocompetent participants allowed for evaluation of baseline presence in a low-risk population, it precludes meaningful conclusions regarding pathogenic relevance and limits extrapolation to high-risk clinical scenarios. Comparative evaluation involving immunocompromised populations would be necessary to better understand the oral ecology and potential reservoirs of mucormycetes.

Additional limitations include potential variability in subgingival sampling despite standardized site selection and instrumentation, as microbial composition may differ between periodontal sites within the same individual [2,3]. The study also did not quantify plaque levels or periodontal inflammatory burden using indices such as plaque index or bleeding on probing, which may have provided further insight into local ecological conditions influencing fungal colonization. Furthermore, the cross-sectional design captures microbial presence at a single time point and does not account for transient or episodic colonization, limiting inference regarding temporal dynamics or causal relationships between periodontal inflammation and fungal presence.

Finally, the investigation focused exclusively on subgingival plaque and did not evaluate other potential oral niches such as saliva, oral mucosa, extraction sockets, or maxillary sinus-adjacent sites, where mucormycetes have been more commonly reported in clinical cases [8,11,13]. Taken together, these limitations indicate that the findings should be interpreted as preliminary and hypothesis-generating. Larger studies incorporating longitudinal designs, molecular detection methods, broader oral sampling, and inclusion of high-risk populations are required to more definitively assess the role of mucormycetes in oral and periodontal environments.

Clinical and research implications

The findings of this exploratory study indicate that cultivable mucormycetes were not detected in the subgingival plaque of immunocompetent individuals with or without periodontitis under the culture conditions employed. From a clinical perspective, this suggests that routine periodontal disease in systemically healthy individuals may not represent an overt or readily detectable reservoir for mucormycetes when assessed using conventional microbiological techniques. This observation is particularly relevant in the post-COVID-19 era, during which increased attention has been directed toward the oral cavity as a potential portal of entry for mucormycosis, especially in medically compromised patients [7,9,11,12]. However, given the opportunistic nature of mucormycetes and their strong association with systemic immune dysregulation, the absence of detection in this low-risk cohort should not be interpreted as evidence of absence in higher-risk populations [10,12,13].

From a periodontal standpoint, these findings reinforce the importance of maintaining periodontal health as part of comprehensive infection prevention strategies. Periodontitis is characterized by a dysbiotic shift in the subgingival microbiome and disruption of host-microbial homeostasis, which may theoretically create a permissive niche for opportunistic microorganisms under favorable systemic conditions [1-4]. Although mucormycetes were not identified in the present study, periodontal inflammation is known to alter local environmental factors such as oxygen tension, nutrient availability, and tissue integrity, all of which may influence microbial colonization patterns in susceptible hosts [3,5]. Consequently, clinicians should exercise heightened caution when managing patients with systemic risk factors such as uncontrolled diabetes mellitus, hematologic malignancies, or immunosuppressive therapy, particularly before invasive dental procedures that compromise mucosal barriers [6,9,10].

The study also highlights important research implications regarding diagnostic methodology. The absence of cultivable mucormycetes underscores the inherent limitations of culture-based techniques for detecting rare or low-abundance fungal organisms within complex oral biofilms. Previous reports indicate that Mucorales may be present in dormant, nonviable, or nonculturable states, which are unlikely to be detected using standard Sabouraud dextrose agar alone [10,12]. Future investigations should therefore incorporate molecular approaches such as polymerase chain reaction or ribosomal RNA-based sequencing to improve sensitivity and enable more accurate characterization of the oral mycobiome [12].

Inclusion of high-risk cohorts represents a critical direction for future research. Mucormycosis predominantly affects individuals with impaired host defenses, including those with diabetes mellitus, hematologic malignancies, or postviral immune dysregulation, and several case reports have documented periodontal or oral involvement in such patients [6-11,13]. Comparative studies involving immunocompetent and immunocompromised individuals may help determine whether subgingival plaque serves as a transient reservoir or a secondary site of colonization under specific systemic conditions.

Finally, longitudinal study designs with repeated sampling over time would allow detection of episodic or transient fungal colonization that may not be captured at a single time point, particularly during periods of systemic illness or periodontal exacerbation. Expanding sampling to include saliva, oral mucosa, and extraction or surgical sites may further clarify the distribution and clinical relevance of mucormycetes within the oral cavity [5,8,14]. Such integrated approaches could ultimately contribute to improved risk stratification, early detection, and preventive strategies in vulnerable patient populations.

Future perspectives

Future research should involve larger, multicenter cohorts with substantially increased sample sizes to improve the detection of rare fungal organisms and enhance external validity. Incorporation of molecular techniques such as polymerase chain reaction (PCR) or 18S rRNA sequencing would allow identification of nonculturable or low-abundance mucormycetes that may be missed by conventional culture-based methods. Studies focusing on high-risk populations, including individuals with uncontrolled diabetes, post-COVID-19 patients, or those with recent maxillofacial trauma or dental extractions, are essential to evaluate predisposing systemic and local factors. Longitudinal study designs are particularly important, as repeated sampling over time could capture transient or episodic fungal colonization that may not be detectable at a single time point and could help clarify temporal relationships between periodontal inflammation, shifts in the oral microbiome, and fungal emergence. Additionally, broader sampling strategies encompassing saliva, oral mucosa, and subgingival plaque would provide a more comprehensive mapping of mucormycetes’ oral distribution. Collectively, these approaches may clarify whether subgingival plaque serves as a latent reservoir under specific clinical conditions and help inform preventive strategies in both periodontal and systemic health [12,13].

Synthesis and broader context

This study’s negative results do not negate the oral relevance of mucormycetes but suggest that subgingival plaque is an unlikely niche in immunocompetent individuals. Periodontitis research has historically focused on bacteria, yet fungal roles remain underexplored [2]. As opportunistic infections rise, understanding microbial diversity in oral biofilms becomes increasingly important. This study bridges a knowledge gap, offering a foundation for comprehensive investigations into fungal-periodontal interactions.

## Conclusions

This study found no mucormycetes in subgingival plaque from 10 periodontitis and 10 non-periodontitis patients, indicating that immunocompetent individuals may not be at risk for mucormycosis. The results establish a preliminary benchmark, suggesting that mucormycetes do not routinely contribute to periodontal biofilms in immunocompetent individuals. However, limitations, including small sample size, culture-based detection, and a healthy population, temper broad conclusions. The findings underscore the microbial complexity of the oral cavity and the need to explore fungal ecology beyond traditional bacterial-centric paradigms. We recommend larger studies with advanced molecular tools, diverse populations (e.g., immunocompromised patients), and longitudinal designs to definitively assess the role of mucormycetes in oral health and periodontal disease, potentially guiding clinical management of opportunistic fungal infections.
